# Correlations between player positions, trunk stability, and functional athletic performance in adolescent female basketball players

**DOI:** 10.1186/s13102-025-01445-3

**Published:** 2025-11-27

**Authors:** Ian-Ju Liang, Linda L. Lin

**Affiliations:** 1 School of Medicine, University of Dundee, Dundee, UK; 2 Institute of Physical Education, Health &Leisure Studies, National Cheng Kung University, Tainan, Taiwan

**Keywords:** Player positions, Core stability, Functional performance, Athletic performance, Female basketball players

## Abstract

**Background:**

Functional performance and physical demands vary by playing position in basketball, with some evidence suggesting differences in speed, agility, and endurance among adolescent athletes. However, the relationship between position-specific roles and measures of trunk stability, balance, and proprioception in female youth players remains underexplored. This study examined the relationships between player positions, trunk stability, and functional performance in adolescent female basketball players.

**Methods:**

Sixty adolescent female basketball players (28 forwards, 16 centres, 16 guards, mean age = 14.9 ± 1.74 years) were assessed on a 20-metre sprint, 20-metre shuttle run (cardiorespiratory endurance), balance, ankle proprioception, and a modified double-leg lowering task (trunk stability). Differences across playing positions (guard, forward, centre) were analysed using analysis of covariance, controlling for height and weight.

**Results:**

Cardiorespiratory endurance significantly differed by position (*p* = .011), with centres exhibiting lower maximal oxygen consumption (51.6 ± 4.5 ml/kg/min) than forwards (53.5 ± 4.8 ml/kg/min). Sprint times showed no statistically significant differences, although forwards were slightly faster (3.72 ± 0.20 s) than centres (3.83 ± 0.16 s). No significant differences were observed across positions in trunk stability, balance, or ankle proprioception.

**Conclusions:**

This study shows that cardiorespiratory endurance is position-dependent in adolescent female basketball players, whereas trunk stability, balance, and ankle proprioception do not vary by position. These findings support the development of targeted training strategies based on playing position and highlight the need for future research to determine whether such functional differences contribute to injury risk or influence performance outcomes, and how early intervention may optimise long-term athlete development.

## Introduction

Basketball, renowned for its dynamic nature and strategic intricacies, demands a multifaceted skill set and a diverse range of physical attributes from its players [1]. As is known, basketball is a high speed, intense, physical game that demands strength, power, speed, balance, coordination, and dribble control based on specific on-court movements such as jump shooting, shot blocking, fast breaks, and rebounding [2, 3]. Studies have found that integrating strength and power training as well as balance and proprioception training into the overall strategy is crucial for enhancing on-court performance, with additional benefits of reducing injury risk [4]. Among the factors influencing performance, the specific playing position of an athlete emerges as a pivotal determinant, shaping their functional capabilities on the court [5]. While involvement in youth sports such as basketball provides numerous potential advantages for children and adolescents [6], there is a concern that an overemphasis on intense, sport-specific training and competition during early stages of development could hinder an athlete’s capacity to develop transferable functional skills and potentially elevate the risk of burnout and overuse injuries [7]. Understanding the relationship between player positions and functional athleticism in this cohort is therefore important for devising tailored training programmes that optimise on-court performance and safeguard against potential injuries [8].

The sport of basketball has experienced significant transformations over the last decade [9]. As basketball players assume distinct roles on the court, be it as forwards, centres, or guards, their physiological demands and skill requirements undergo notable variations [8, 10]. The performance expectations for each position are unique, emphasising specific attributes that contribute to the overall success of the team. Forwards, often relied upon for fast breaks and scoring, necessitate exceptional speed, agility, jumping ability, especially for lay-up and jump shooting [11, 12]. Centres, acting as anchors in both offense and defence, require robust trunk stability, rebounding ability, and efficient cardiorespiratory endurance [13]. Yet centres spend significantly less time engaging in high-intensity activities compared to guards and forwards [14]. Guards, managing plays and defending against opponents, benefit from a harmonious blend of speed, agility, and precise proprioception for the needs of defensive shuffle and sudden changes of directions [11, 12, 15]. Each position therefore carries distinct physiological and technical requirements. Accordingly, basketball players must develop strong functional athletic abilities (i.e., speed, agility, strength, and power) tailored to positional demands to optimise performance [16]. The dynamic demands of basketball, including the high-intensity, intermittent and short recovery periods nature of the game, emphasise the necessity to assess players’ physical performance for tailored training programmes [17]. In particular, in adolescent female basketball players aged 12–18 years, a phase marked by rapid physical development and skill acquisition, exploring the correlations between player positions and key functional attributes becomes particularly significant [16].

Numerous studies have highlighted significant variations among playing positions concerning body size, speed, agility, vertical jump, and maximum oxygen consumption [18, 19]. Sampaio, Janeira emphasised the importance of considering diverse contributions and playing positions when evaluating game performance in elite male basketball players. Similarly, García, Vázquez-Guerrero compared the physical demands across game quarters and playing positions in professional male basketball players and emphasised the need for tailored training drills to optimise game performance including incorporating basketball-specific tasks with appropriate work-to-rest ratios and position-specific training during the off-season to address unique player demands. Köklü, Alemdaroğlu found that elite male Turkish basketball players, specifically centres, exhibited lower agility, while guards demonstrated higher cardiorespiratory endurance and speed [20]. Delextrat and Cohen observed that guards outperformed forwards and centres in speed, strength, jumping ability, and agility among adult female basketball players [21], aligning with findings from another study investigating physical characteristics in female basketball players aged 15–17 [22]. A recent systematic review reinforced these positional distinctions, showing that guards generally perform the greatest running volumes with frequent accelerations, forwards display higher levels of high-speed activity, and centres contribute more to rebounding and scoring [23]. These studies highlight the importance of tailoring fitness programmes based on specific playing positions in professional basketball.

However, most existing research on the relationship between physical function and player positions has predominantly focused on male and/or female adult cohorts. There is a need for further investigation to address a similar topic within adolescent female basketball players. This study seeks to delve into the intricate relationship between player positions, trunk stability, and overall functional athletic performance in a cohort of female basketball players aged between 12 and 18 years. By examining the functional profiles associated with different positions, we aimed to uncover areas of strength and potential improvement for each player.

## Materials and methods

### Study design

This investigation was designed to address the imperative need for understanding the nuanced variations in functional performance among different basketball player positions. The overarching goal was to inform the development of customised training programmes tailored to enhance on-court performance in female basketball players aged 12–18 years. The assessed basketball-specific functional performance aspects included core stability, balance and ankle proprioception, speed, and cardiopulmonary endurance. The protocol and purpose of this study were both approved by the Human Experiment and Ethics Committee of National Cheng Kung University Hospital (reference number: A-ER-108–537).

### Participants

Sixty female basketball players, with an average age of 14.9 ± 1.74 years, were recruited for this study. All participants were ranked among the top four in national junior and senior high school basketball tournaments. The sample was diversified, comprising 28 forwards, 16 centres, and 16 guards. Players were categorised into these three traditional basketball positions to reflect distinct functional and physiological demands associated with each role [18, 23]. While some studies classify athletes more broadly into inside and outside players, this two-group system may obscure meaningful differences between forwards and centres [23], particularly in adolescent cohorts where role specialisation is already established.

With an average height of 166.9 ± 8.1 cm and a weight of 59.6 ± 7.9 kg, the participants aimed to provide a comprehensive representation of the age and physical characteristics inherent to adolescent female basketball players. Exclusion criteria included musculoskeletal injuries within three months preceding assessments and a history of vestibular dysfunction or concussion. Prior to engaging in the study, all participants willingly provided informed consent. For participants younger than 16 years of age, informed consent was additionally obtained from their parents or legal guardians.

### Procedures

All participants underwent core stability and functional athletic performance assessments. Outcome measures included the modified double leg lowering task (MDLL) for evaluating trunk stability [24], as well as assessments in athletic performance domains, namely speed, cardiopulmonary endurance, balance and ankle proprioception.

In particular, the MDLL level was determined using a stabiliser pressure biofeedback unit, gauging subjects’ trunk stability [25]. The scoring criteria for the MDLL are primarily based on the hip joint angle, representing the angle between the floor and participants’ legs (i.e., hip flexion angle). Scores are categorized as follows: 70–90 degrees as 5, 60–75 degrees as 6, 45–60 degrees as 7, 30–45 degrees as 8, 15–30 degrees as 9, and 0–15 degrees as 10. A higher score indicates better trunk stability. The MDLL assessment has demonstrated high reliability (γ = 0.95) and is considered a valid measure of trunk stability [26].

Speed was assessed through 20-metre sprint tests using a dash timing gate system with laser detection. Participants started from a stationary position and were instructed to sprint as fast as possible. Each participant performed two trials with a 5-minute rest interval, and the fastest time was recorded. The reliability for 20-metre sprints in youth athletes has been reported as excellent, with intraclass correlation coefficients of 0.89–0.95 [27].

Cardiopulmonary endurance was measured using 20-metre shuttle run tests [28], a validated and reliable measure of aerobic capacity. Its reliability has been reported as excellent (γ = 0.95) [29]. Participants ran back and forth between two lines 20 m apart in time with auditory cues, with each stage increasing in speed (starting at 7.5 km·h⁻¹, second stage at 8.7 km·h⁻¹, and increasing by 0.5 km·h⁻¹ per stage) [30, 31]. The test ended when a participant failed to reach the line twice consecutively before the beep. VO₂ max was then estimated from shuttle run performance using the formula: VO₂ max (ml/kg/min) = 5.857 × stage speed (km/h) − 19.458 [32].

Balance and ankle proprioception were assessed using the BioSway balance system (Biodex BioSway, Portable, Inc., Shirley, New York), which incorporated the modified Clinical Test of Sensory Integration of Balance (mCTSIB) protocol with four task conditions (i.e., open-eye firm surface, close-eye firm surface, open-eye foam surface, and close-eye foam surface). The Sway Index (SI) recorded by the system was calculated, with a lower SI indicating superior balance and ankle proprioception. Its reliability has been reported as moderate to good, with intraclass correlation coefficient values ranging from 0.58 to 0.81 [31].

### Statistical analyses

Data analysis was performed using SPSS version 28.0. Descriptive statistics were presented as means and standard deviations. A one-way ANOVA was employed to assess baseline differences among different positions. Subsequently, an analysis of covariance (ANCOVA) was applied to discern position-related differences in various dependent variables. Height and weight served as covariates to enhance result precision, accounting for potential confounding factors. This statistical approach aimed to unravel distinct functional profiles associated with various playing positions, with a significance level set at *p* <.05.

### Patient and public involvement

Patients and members of the public were not involved in the design, conduct, reporting, or dissemination of this research. This study focused on physiological and performance-based assessments in healthy adolescent athletes within a controlled sports setting. As such, the research questions, methods, and outcomes were primarily driven by scientific and performance-related considerations. Future research exploring injury risk, long-term health outcomes, or athlete well-being may benefit from involving young athletes and coaches in the design process.

## Results

Table 1 presents the demographic characteristics of the participants stratified by playing positions, namely Forwards, Centres, and Guards. No statistically significant differences were observed in age among the three groups (*p* =.668). However, significant differences were found in height (*p* <.001) and weight (*p* =.001), with Centres having a taller stature and higher weight compared to Forwards and Guards.

**Table 1:** Participant characteristics

Characteristics	Forwards (*n* = 28)	Centres (*n* = 16)	Guards (*n* = 16)	*p*-value
Age (years)	14.79 ± 1.77	15.06 ± 1.44	14.5 ± 2.03	0.668
Height (cm)	166.21 ± 4.56	174 ± 4.34	159.88 ± 5.03	< 0.001*
Weight (kg)	58.57 ± 6.56	64.06 ± 6.36	54.88 ± 7.56	0.001*

*Significant at *p* <.05

Regarding functional performance measures, no significant differences were observed in 20-metre sprint velocity (*p* =.192). Nevertheless, Centres (3.83 ± 0.16 seconds) demonstrated a trend of lower speed ability compared to Forwards (3.72 ± 0.2 seconds). Cardiorespiratory endurance significantly differed among player positions (*p* =.011) with a large effect size (R² =.207). Specifically, Centres exhibited significantly lower maximal oxygen consumption compared to Forwards (*p* =.004). Core stability did not show significant differences among the groups (*p* =.761). Balance and ankle proprioception assessments on open-eye firm surface (*p* =.200), close-eye firm surface (*p* =.738), open-eye foam surface (*p* =.704), and close-eye foam surface (*p* =.929) also revealed no significant variations. However, participants’ overall balance and ankle proprioception performance was poorer than typical adolescent’ norms (see Table 2), especially in open-eye foam and close-eye foam conditions; the lower the Stability Index (SI), the better the balance and ankle proprioception.

**Table 2:** Adolescent m-CTSIB normative data

Conditions	Open-eye Firm Surface	Close-eye Firm Surface	Open-eye Foam Surface	Close-eye Foam Surface
Sway Index	0.48	0.66	0.75	1.87

The lower Sway Index indicates superior balance and ankle proprioception

These norms represent populations aged between 13–18

**Fig. 1:**
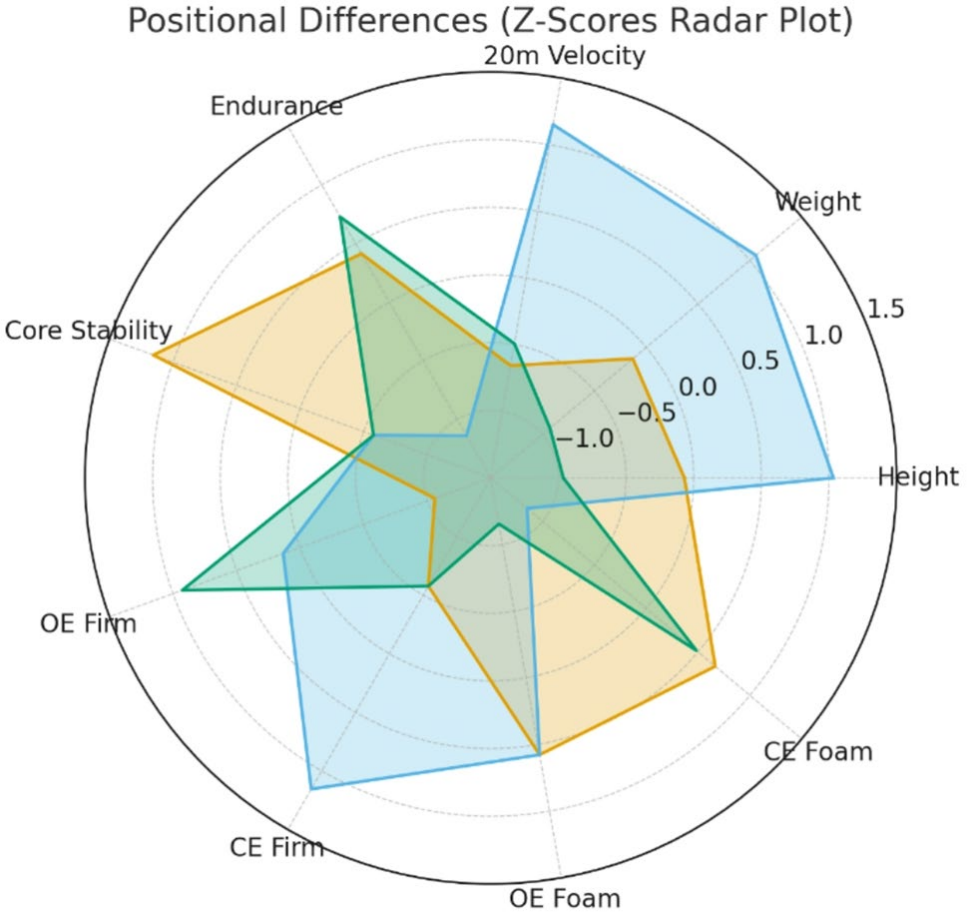
Positional differences in height, weight, and physical performance profiles (Z-scores)

Table 3 displays mean and standard deviation values for Forwards, Centres, and Guards, along with *p*-values and Partial Eta Squared (R²), presenting the results of various functional performance measures across different playing positions. Figure 1 provides a visual overview of the characteristics (i.e., height and weight) and physical performance profiles across the three positional groups.

**Table 3:** Functional performance measures across player positions (mean ± SD, *p*-values, and effect sizes)

Measures	Forwards (*n* = 28)	Centres (*n* = 16)	Guards (*n* = 16)	*p*-value	Partial Eta Squared (*R*²)
20-metre Velocity (sec)	3.72 ± 0.2	3.83 ± 0.16	3.73 ± 0.14	0.192	0.073
Endurance (ml/kg/min)	53.46 ± 4.82	51.63 ± 4.52^‡^	53.83 ± 4.39	0.011*	0.207
Core Stability (grade)	5.32 ± 0.55	5.31 ± 0.6	5.31 ± 0.48	0.761	0.020
Open-eye Firm Surface	0.45 ± 0.18	0.51 ± 0.21	0.55 ± 0.25	0.200	0.110
Close-eye Firm Surface	0.56 ± 0.2	0.58 ± 0.22	0.56 ± 0.2	0.738	0.050
Open-eye Foam Surface	0.98 ± 0.34	0.98 ± 0.28	0.94 ± 0.35	0.704	0.182
Close-eye Foam Surface	2.1 ± 0.4	2 ± 0.49	2.09 ± 0.74	0.929	0.076

*Significant at *p* <.05

^‡^Significant group differences compared to the Forwards. The parameter estimate for Centres is −7.131, indicating a significantly lower endurance (VO_2_max ml/kg/min) compared to Forwards (*p* =.004)

Endurance (ml/kg/min) is the estimated VO₂max derived from the 20-m shuttle run test

Partial eta squared (R²) values were interpreted as small (≈ 0.01), medium (≈ 0.06), or large (≥ 0.14) [33]

Z-scores standardised all variables across positions. Note: higher 20 m sprint times and higher OE/CE balance scores (SI) indicate slower speed and poorer balance/ankle proprioception. Centres (blue) are generally taller and heavier with slower sprint performance, Forwards (gold) show higher core stability, and Guards (green) demonstrate better endurance. This figure summarises positional differences in height, weight, speed, endurance, core stability, and balance/ankle proprioception. OE = open-eye; CE = closed-eye.

## Discussion

In examining the physical characteristics of adolescent female basketball players across different positions, significant variations in height and weight were identified, with centres displaying a taller stature and higher weight compared to forwards and guards. These findings align with the expectations associated with specific player roles, emphasising the importance of distinct physiological attributes for each position [18]. Nevertheless, the lack of significant variation in age across playing positions helps ensure that the functional performance differences observed are less likely to be confounded by age-related factors.

Indeed, understanding the intricate dynamics associated with different playing positions is imperative for tailored training programmes as basketball is a team sport characterised by intermittent play, involving a mix of high-intensity actions and intervals of lower intensity or recovery [34]. Despite its substantial cardiorespiratory demands and reliance on aerobic metabolism, achieving success at the elite level in basketball is primarily contributed by rapid-driven sport-specific movements [35]. In the discussion of these results, we will delve into key aspects, including speed, cardiorespiratory endurance, core stability, and balance and ankle proprioception respectively.

### Sprinting speed

The observed trend of faster speed in forwards compared to centres aligns with the positional expectations within the sport. Forwards primarily tasked with fast breaks, lay-up and scoring, demonstrated superior speed abilities. Centres, in contrast, are typically larger athletes with roles emphasising post play, rebounding, and defence, which may partially explain their slower sprint times [13]. Previous studies have similarly reported that centres display lower running velocities and fewer changes of direction than other positions during games [8, 20] and lower velocities than forwards [13, 18]. While these observations reflect typical positional characteristics, evolving strategies and playing cultures in modern basketball may increasingly require centres to engage in faster transitional play [36]. Therefore, improving speed could potentially benefit centres.

Nevertheless, our cohort’s 20-metre sprint times were comparable to those reported for adolescent female basketball players of similar competitive levels [37], but slower than values observed in elite senior players [38], highlighting the developmental gap. This reinforces the importance of structured speed training in this age group. The trend also suggests potential differences in the speed demands of various positions, with further investigation including larger sample size and wider populations warranted to elucidate these nuances.

### Cardiorespiratory fitness

The significant difference in cardiorespiratory endurance among player positions, with centres exhibiting lower maximal oxygen consumption compared to forwards, demonstrates the unique physiological demands of different roles. Similarly, previous studies, using different tests to determine the cardiorespiratory fitness (e.g., maximal treadmill tests, maximal cardiopulmonary cycle ergometer exercise tests, and total distance covered during the game), indicated that centres had lower cardiorespiratory endurance than forwards and guards [11, 18]. Centres, acting as anchors in both offense and defence, tending to remain in more static positions than other players may explain this result. Despite these results, our cohort’s estimated VO₂ max was within the range reported for basketball players aged 17–25 [39, 40], suggesting that aerobic endurance continues to develop during adolescence and may influence the ability to sustain high-intensity performance across game quarters [41]. Furthermore, variations in VO₂ max during adolescence are influenced by biological maturation, training experience, and body composition, highlighting the importance of monitoring aerobic capacity to optimise performance in young basketball players [42].

### Core stability

While no significant differences were found in core stability among player positions, the importance of trunk stability for overall athletic performance cannot be understated. In one hand, this suggests that within the context of adolescent female basketball, players across positions may share a relatively even level of trunk stability. Core muscles, extending from the thoracic spine to the hips, play a crucial role in fundamental movements [43]. A recent study involving male adolescent basketball players showed that core strength training enhanced balance, agility, and dribbling skills [44]. It has been, indeed, shown that trunk function is not only related to the degree of physical fitness but also important for the balance and stability of the whole body [45, 46]. Moreover, a lot of benefits of core stabilisation have been touted, from improving athletic performance to preventing injuries [47]. Consequently, having higher scores on the modified double-leg lowering task indicate better trunk stability, which may contribute to better power transmission and injury prevention for players in all positions [48]. On the other hand, the lack of statistical significance may be attributed to the comprehensive fitness levels inherent to elite adolescent female basketball players (i.e., ceiling effect) [46]. Future studies could explore the nuances of core stability in relation to specific game scenarios and playing positions.

### Balance and ankle proprioception

The absence of significant differences in balance and ankle proprioception assessments indicates that these attributes may be universally developed across playing positions. However, our participants’ SI values were poorer than normative values for age-matched adolescents, particularly under foam conditions, indicating below-average balance performance despite their competitive level. Research has indicated that individuals participating in sports involving jumping and turning, such as basketball, volleyball, or soccer, commonly experience functional ankle instability [49]. As the matter of fact, this result may be due to overuse or repetitive ankle movements during running, jumping, and landing, imposing considerable stress on the lower limbs and leading to ankle instability [50]. A systematic review of balance training in basketball players demonstrated that targeted proprioceptive and balance interventions significantly enhance physical fitness and on-court skills [51]. Accordingly, targeted balance and ankle stability training is essential to enhance on-court overall functions and lower extremity control abilities in this cohort.

Altogether, the study’s findings have practical implications for coaches and fitness trainers working with adolescent female basketball players. Tailored training interventions can be devised to address the specific needs of players based on their positions. Recognising the specific performance requirements for each position is imperative for developing targeted training interventions aimed at enhancing individual and team performance. The importance of such tailored training programmes lies not only in elevating individual player capabilities but also in optimising team dynamics [4]. For instance, a forward with enhanced speed and endurance can capitalise on fast breaks and dynamic plays, creating scoring opportunities [52]. A centre with improved trunk stability can withstand physical challenges in the paint, contributing to a formidable defence [53]. Guards with heightened proprioception can navigate the court with precision, facilitating effective playmaking [54].

Based on the results of the current study, training professions can utilise speed training for centres, cardiorespiratory fitness programmes for forwards, and balance based as well as ankle proprioception enhancement for all players that could contribute to a more holistic approach to player development for a whole team.

### Limitations

Despite the valuable insights gained, this study has several limitations. Firstly, the results, based on a small sample size and specific player levels, cannot be generalised to wider basketball populations. Additionally, the cross-sectional design provides a snapshot of functional performance, and longitudinal studies could offer a more comprehensive understanding of player development. Factors beyond height and weight, such as playing experience and skill proficiency, were not explored in depth. Moreover, the potential impact of biological maturation on functional performance and fat-free mass should be acknowledged. Recent studies using the force-velocity test in youth basketball populations have shown that anaerobic peak power is strongly associated with body size and fat-free mass, with maturation status further explaining inter-individual variance in performance [55, 56]. Thus, differences in sprint or power-related measures may partly reflect maturational stage and muscle mass, rather than solely positional demands. Future research should incorporate assessments of maturation to better contextualise functional outcomes in adolescent basketball players. Continuous research in this domain is crucial. Future studies could explore additional performance measures, consider the impact of game-specific scenarios, and incorporate longitudinal designs for a more comprehensive understanding of the dynamic interplay between player positions and functional athleticism. Accordingly, it would be worth exploring the correlations between players’ real-time dedication during the game and their physical function and playing positions to gain insight into the relationships between actual playing time and physical demands across three different playing positions. Researchers can conduct more high-quality studies to gather knowledge on the modern basketball trend, where studies found that professional players spent a significant amount of time (38–77%) in low-intensity activities during the games [10, 35].

Indeed, this research not only adds to the scientific discourse on sports performance but also provides practical insights for coaches, fitness trainers, athletic trainers, and sports practitioners working with adolescent female basketball players. As the dynamics of the basketball game continue to evolve, understanding and adapting training strategies to the nuanced requirements of different positions become essential for the holistic development of young athletes.

## Conclusions

This study explored the associations between player position, trunk stability, and functional performance in adolescent female basketball players. The findings showed that while height and weight differed between positions, functional measures such as sprint performance, trunk stability, and balance did not vary substantially across groups. The only significant positional difference was observed in cardiorespiratory endurance, where centres demonstrated lower VO₂ max compared to forwards. These results indicate that, while training requirements are generally consistent across positions, enhancing aerobic capacity in centres could help meet the physical demands of modern play. Furthermore, balance and proprioceptive scores were lower than adolescent norms across all positions, emphasising a need for targeted interventions regardless of playing role. Overall, the study contributes to the understanding of functional demands in adolescent female basketball and provides practical guidance for designing evidence-based, position-aware training programmes.

## Data Availability

The data are available upon request from the authors.
